# Supplementary material for: Bruton’s Tyrosine Kinase Inhibitors Ibrutinib and Acalabrutinib Counteract Anthracycline Resistance in Cancer Cells Expressing AKR1C3
Source: Cancers (Basel). 2020 Dec 11;12(12):3731. doi: 10.3390/cancers12123731 (PMC7764606; doi:10.3390/cancers12123731)
Supplement: Supplementary file 1 [file cancers-12-03731-s001.pdf]

Table S1. Synergism between BTK-inhibitors and Dau rely on AKR1C3 expression in HCT116 cells.

BTK-inhibitor	Concentration (µM)	Dau (µM)	Fraction affected (Fa %)	Combination index (CI)	Dose reduction index for Dau
Ibrutinib			HCT116 - EV		
	5	0.25	58.13 ± 12.2	0.64 ± 0.08	1.65 ± 0.31
		0.50	77.56 ± 6.90	0.75 ± 0.09	1.35 ± 0.17
		0.75	89.66 ± 1.46	0.68 ± 0.01	1.47 ± 0.02
		1.00	89.82 ± 0.70	0.91 ± 0.13	1.11 ± 0.15
	10	0.25	58.56 ± 7.79	0.66 ± 0.02	1.65 ± 0.14
		0.50	81.92 ± 4.25	0.65 ± 0.03	1.55 ± 0.10
		0.75	91.71 ± 0.70	0.60 ± 0.09	1.68 ± 0.25
		1.00	89.89 ± 1.09	0.91 ± 0.15	1.12 ± 0.18
			HCT116 - C3		
	5	0.25	37.42 ± 3.62	0.64 ± 0.08	1.80 ± 0.07
		0.50	71.44 ± 3.52	0.33 ± 0.04	3.06 ± 0.44
		0.75	81.47 ± 2.11	0.31 ± 0.04	3.29 ± 0.50
		1.00	88.11 ± 1.40	0.26 ± 0.05	3.86 ± 0.69
	10	0.25	47.30 ± 4.69	0.46 ± 0.05	2.55 ± 0.24
		0.50	78.19 ± 0.39	0.25 ± 0.01	4.10 ± 0.00
		0.75	89.25 ± 1.68	0.18 ± 0.04	5.71 ± 1.25
		1.00	92.29 ± 0.74	0.18 ± 0.03	5.78 ± 1.05
Acalabrutinib			HCT116 - EV		
	5	0.25	57.98 ± 10.7	0.64 ± 0.07	1.59 ± 0.16
		0.50	76.14 ± 4.55	0.80 ± 0.02	1.26 ± 0.03
		0.75	88.86 ± 2.18	0.73 ± 0.05	1.38 ± 0.09
		1.00	89.81 ± 0.07	0.93 ± 0.17	1.10 ± 0.20
	10	0.25	58.22 ± 6.27	0.64 ± 0.01	1.60 ± 0.00
		0.50	81.17 ± 2.72	0.68 ± 0.05	1.48 ± 0.11
		0.75	91.66 ± 0.08	0.62 ± 0.12	1.65 ± 0.31
		1.00	90.59 ± 0.95	0.88 ± 0.12	1.15 ± 0.15

			HCT116 - C3		
	5	0.25	34.59 ± 5.75	0.76 ± 0.21	1.38 ± 0.37
		0.50	68.43 ± 1.41	0.42 ± 0.06	2.38 ± 0.34
		0.75	79.88 ± 0.40	0.38 ± 0.05	2.67 ± 0.32
		1.00	86.61 ± 1.29	0.33 ± 0.03	3.05 ± 0.28
	10	0.25	40.91 ± 11.4	0.63 ± 0.28	1.77 ± 0.75
		0.50	77.47 ± 0.69	0.28 ± 0.03	3.53 ± 0.33
		0.75	87.59 ± 1.02	0.23 ± 0.03	4.40 ± 0.51
		1.00	91.62 ± 0.29	0.21 ± 0.04	4.87 ± 1.00

Values are expressed as the means ± SD from six independent experiments.

Table S2. Synergism between BTK-inhibitors and Dau on A549 cells.

BTK-inhibitor	Concentration (µM)	Dau (µM)	Fraction affected (Fa %)	Combination index (CI)	Dose reduction index for Dau
**Ibrutinib**	1	0.01	21.92 ± 2.37	0.45 ± 0.03	2.78 ± 0.10
		0.05	31.67 ± 3.47	0.56 ± 0.13	1.95 ± 0.31
		0.25	43.16 ± 2.59	0.96 ± 0.05	1.06 ± 0.03
		0.50	71.16 ± 6.46	0.89 ± 0.11	1.14 ± 0.14
		0.75	86.63 ± 0.72	0.72 ± 0.13	1.43 ± 0.25
		1.00	91.12 ± 0.72	0.71 ± 0.11	1.43 ± 0.23
	5	0.01	33.76 ± 1.79	0.41 ± 0.18	4.11 ± 0.30
		0.05	41.12 ± 3.30	0.50 ± 0.14	2.52 ± 0.23
		0.25	54.13 ± 5.98	0.75 ± 0.02	1.41 ± 0.09
		0.50	85.67 ± 2.02	0.51 ± 0.04	2.01 ± 0.11
		0.75	91.76 ± 0.78	0.51 ± 0.06	2.01 ± 0.24
		1.00	94.00 ± 0.28	0.54 ± 0.08	1.88 ± 0.28

	10	0.01	37.23 ± 5.99	0.47 ± 0.21	4.51 ± 0.40
		0.05	44.72 ± 5.64	0.51 ± 0.15	2.76 ± 0.18
		0.25	59.78 ± 11.03	0.67 ± 0.07	1.67 ± 0.36
		0.50	88.10 ± 2.98	0.44 ± 0.01	2.33 ± 0.14
		0.75	92.99 ± 1.03	0.45 ± 0.07	2.26 ± 0.34
		1.00	94.03 ± 0.56	0.54 ± 0.11	1.91 ± 0.40
**Acalabrutinib**	1	0.01	18.46 ± 1.06	0.53 ± 0.06	2.47 ± 0.11
		0.05	29.07 ± 6.04	0.62 ± 0.15	1.57 ± 0.53
		0.25	44.39 ± 2.96	0.93 ± 0.08	1.11 ± 0.07
		0.50	70.57 ± 4.30	0.91 ± 0.11	1.12 ± 0.13
		0.75	85.08 ± 0.89	0.78 ± 0.07	1.30 ± 0.11
		1.00	89.45 ± 1.83	0.80 ± 0.14	1.27 ± 0.24
	5	0.01	32.70 ± 2.93	0.47 ± 0.20	4.03 ± 0.28
		0.05	39.64 ± 5.43	0.57 ± 0.19	2.46 ± 0.34
		0.25	53.48 ± 4.57	0.78 ± 0.06	1.40 ± 0.18
		0.50	83.62 ± 1.20	0.56 ± 0.04	1.81 ± 0.13
		0.75	90.21 ± 1.06	0.58 ± 0.08	1.77 ± 0.24
		1.00	92.46 ± 1.24	0.64 ± 0.11	1.61 ± 0.30
	10	0.01	36.02 ± 5.82	0.58 ± 0.34	4.45 ± 0.77
		0.05	43.34 ± 6.07	0.64 ± 0.28	2.71 ± 0.42
		0.25	57.68 ± 5.04	0.75 ± 0.12	1.57 ± 0.23
		0.50	84.46 ± 1.42	0.55 ± 0.05	1.89 ± 0.17
		0.75	90.20 ± 0.79	0.58 ± 0.03	1.75 ± 0.10
		1.00	92.76 ± 1.59	0.62 ± 0.14	1.68 ± 0.40

Figure S1. Complete western blots with densitometry readings/intensity ratio of each band.

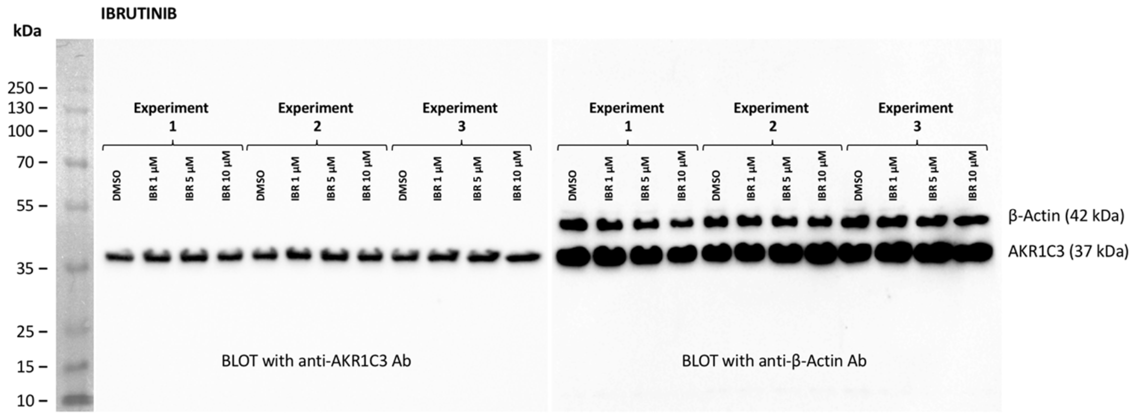

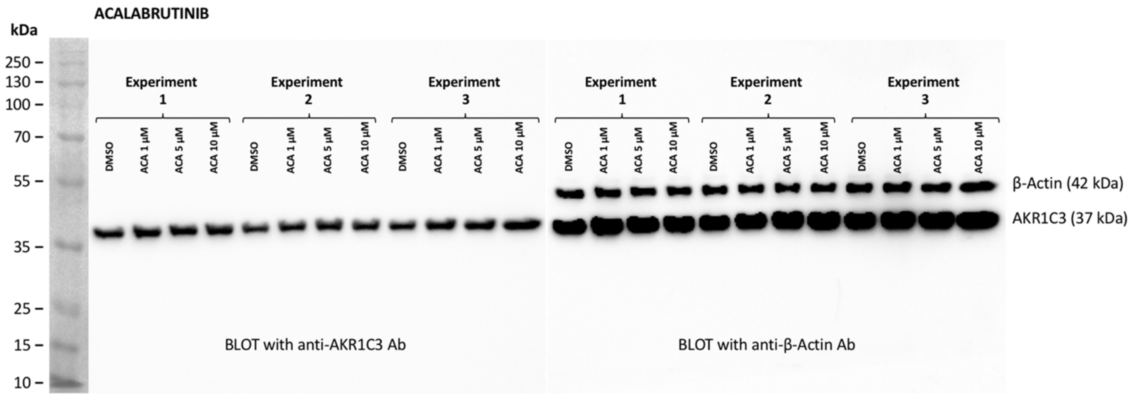

**IBR**	Mean intensity		Intensity ratio
Position	AKR1C3	β-ACTIN	
1	93.851	119.192	0,79
2	109.185	104.395	1,05
3	112.818	87.648	1,29
4	96.713	70.407	1,37
5	97.114	98.220	0,99
6	105.387	98.374	1,07
7	113.512	93.034	1,22
8	103.465	93.807	1,10
9	103.529	128.283	0,81
10	119.513	127.119	0,94
11	124.936	123.950	1,01
12	116.701	121.978	0,96

**ACA**	Mean intensity		Intensity ratio
Position	AKR1C3	β-ACTIN	
1	83.188	84.605	0,98
2	92.440	87.569	1,06
3	91.177	81.064	1,12
4	88.834	76.473	1,16
5	67.500	79.438	0,85
6	75.012	77.505	0,97
7	79.317	82.740	0,96
8	75.645	84.695	0,89
9	70.216	100.914	0,70
10	89.981	108.985	0,83
11	93.604	99.455	0,94
12	116.903	119.129	0,98
